# Search for independent (β/α)_4_ subdomains in a (β/α)_8_ barrel β-glucosidase

**DOI:** 10.1371/journal.pone.0191282

**Published:** 2018-01-16

**Authors:** Vitor M. Almeida, Maira A. Frutuoso, Sandro R. Marana

**Affiliations:** Departamento de Bioquímica, Instituto de Química, Universidade de São Paulo, São Paulo, SP, Brazil; University of Colorado Anschutz Medical Campus, UNITED STATES

## Abstract

Proteins that fold as (β/α)_8_ barrels are thought to have evolved from half-barrels that underwent duplication and fusion events. The evidence is particularly clear for small barrels, which have almost identical halves. Additionally, computational calculations of the thermodynamic stability of these structures in the presence of denaturants have revealed that (β/α)_8_ barrels contain two subunits or domains corresponding to half-barrels. Hence, within (β/α)_8_ barrels, half-barrels are self-contained units. Here, we tested this hypothesis using β-glucosidase from the bacterium *Thermotoga maritima* (bglTm), which has a (β/α)_8_ barrel structure. Mutations were introduced to disrupt the noncovalent contacts between its halves and reveal the presence of two domains within bglTm, thus resulting in the creation of mutants T1 (containing W12A and I217A mutations) and T2 (containing W12A, H195A, I217A and F404A mutations). Mutants T1 and T2 were properly folded, as indicated by their fluorescence spectra and enzyme kinetic parameters. T1 and wild-type bglTm were equally stable, as shown by the results of thermal inactivation, differential scanning fluorimetry and guanidine hydrochloride denaturation experiments. However, T2 showed a first-order inactivation at 80°C, a single melting temperature of 82°C and only one transition concentration (*c*_50_) in 2.4 M guanidine hydrochloride. Additionally, T1 and T2 exhibited a cooperative denaturation process that followed a two-state model (*m*-values equal to 1.4 and 1.6 kcal/mol/M, respectively), similar to that of wild-type bglTm (1.2 kcal/mol/M). Hence, T1 and T2 each denatured as a single unit, although they contained different degrees of disruption between their halves. In conclusion, bglTm halves are equivalent in terms of their thermal and chemical stability; thus, their separate contributions to (β/α)_8_ barrel unfolding cannot be disentangled.

## Introduction

The (β/α)_8_ barrel is the most common protein fold among enzymes. Because of their prevalence, (β/α)_8_ barrels have been proposed to have arisen early in protein evolution [1–4]. The current hypothesis is that (β/α)_8_ barrel proteins evolved from a half-barrel (a (β/α)_4_ unit) that underwent duplication and fusion events [5]. This hypothesis is supported by studies examining several (β/α)_8_ barrel proteins. A compelling illustration is the imidazole glycerol phosphate synthase (HisF) from *Thermotoga maritima*. According to the HisF crystallographic structure, this protein is composed of two superimposable halves. In addition, these (β/α)_4_ units have been produced as isolated recombinant proteins, which if co-refolded or alternatively co-expressed, result in a functional 1:1 complex [6]. Convincing indications of the participation of (β/α)_4_ units in the assembly of the (β/α)_8_ barrels were also observed in studies of the folding of the rabbit muscle triosephosphate isomerase (TIM) enzyme [7]. The C-terminal half of TIM folds faster, producing an intermediate, and then the N-terminal half adopts a native structure. An analysis of *S*. *cerevisiae* TIM proteins in which the native order of the β/α units were shuffled indicated that half-barrel association is the final phase in the folding of (β/α)_8_ barrels [8]. Experiments examining the chemical denaturation of the α-subunit of the tryptophan synthase from *E*. *coli* (αTS) showed that unfolding follows a two-transition process in which the denaturation of the N-terminal half corresponds to the first transition [9]. Finally, a segment corresponding to a (β/α)_4_ unit functions as a precursor in the folding of the N-(5’-phosphoribosyl)anthranilate isomerase (TrpF) from *E*. *coli* [10].

A soluble and stable (β/α)_8_ barrel has been produced by fusing two identical half-barrels from HisF [11, 12]. Later, a catalytically active form of this symmetrical barrel was obtained [13]. These experiments imitate the hypothetical evolutionary route leading to the formation of (β/α)_8_ barrels. A further step in the reconstruction of barrels from halves was the production of active (β/α)_8_ barrels by combining half-barrels derived from distantly correlated glycoside hydrolases [14]. In fact, the assembly of (β/α)_8_ barrels from (β/α)_4_ units applies to any barrel, not only the symmetrical barrels.

Interestingly, the flexibility of the (β/α)_8_ barrels to be built from subdomains not only facilitates the production of synthetic proteins, as described above, but may also contribute to the diversity of proteins encoded in the human genome through alternative splicing events that produce functional fragments of (β/α)_8_ barrel-containing proteins [15].

From the thermodynamic perspective, a protein domain is a “self-contained unit” whose cooperative unfolding depends only on its internal interactions [16]. Hence, a protein domain unfolds with the same cooperativity when it is isolated or located within the protein. The unfolding cooperativity is related to the *m*-values determined in chemical denaturation experiments. Thus, (β/α)_8_ barrels are actually formed by two domains that correspond to their halves, i.e., (β/α)_4_ units [16]. Interestingly, using a completely different approach based on graph theory, the simplest protein segment that was shown to be recombined to produce chimeric (β/α)_8_ barrels is a half-barrel [17]. In addition, this approach has been used to produce a functional β-glucosidase, a (β/α)_8_ barrel protein, by combining halves from proteins classified in different life kingdoms [17]. These observations are consistent with the hypothesis of (β/α)_8_ barrel evolution [1].

The aforementioned experimental evidence indicates that half-barrels are self-contained units within (β/α)_8_ barrels. Theoretically, this hypothesis should be valid for any type of (β/α)_8_ barrel, independent of its size and internal symmetry.

Here, we tested this hypothesis using a β-glucosidase from the bacterium *Thermotoga maritima* (bglTm; 446 amino acid residues; PDB ID: 1OIN) [18]. The bglTm protein has a (β/α)_8_ barrel structure, similar to all members of the glycoside hydrolase family 1 [19]. Actually, glycoside hydrolases are the largest group of enzymes that adopt this fold [3]. Nevertheless, in contrast to HisF [6], the bglTm halves do not have any detectable similarity. We previously investigated the enzyme activity of isolated halves of a distinct β-glucosidase [20]. However, in contrast to our previous study and the studies mentioned above, which analyzed (β/α)_8_ barrel fragments, in the present study, we utilized an alternative approach with the aim of revealing the putative concomitant presence of two domains within an integral (β/α)_8_ barrel. We introduced mutations to disrupt the noncovalent contacts between its halves and probed the thermal and chemical stability of these mutant (β/α)_8_ barrels.

## Materials and methods

### Expression and purification of the wild-type and mutant bglTm proteins

The DNA segments encoding the wild-type and mutant bglTm proteins were produced by GenScript (Piscataway, NJ, US) and cloned into the pLATE51 expression vector (Thermo Scientific, Waltham, MA, US). The primers used in the amplification and cloning steps were 5’-ggtgatgatgatgacaagaacgtgaaaaagttccctgaaggattcc-3’ and 5’-ggagatgggaagtcattatcagtcttccagaccgttgtttttaac-3’. Recombinant pLATE51 vectors encoding bglTm were propagated in XL1 Blue cells (Agilent, Santa Clara, CA, US) and extracted with a Wizard Plus SV Miniprep DNA Purification System (Promega, Madison, WI, US). BL21(DE3) cells (Merck Millipore, Billerica, MA, US) transformed with the recombinant pLATE51 vectors were cultivated in Luria broth (500 mL) containing ampicillin (50 μg/mL) at 37°C with shaking at 150 rpm until the culture reached an optical density at 600 nm in the range 0.4–0.6. Then, protein expression was induced with 1 mM isopropyl β-thio-galactopyranoside (IPTG) for 24 h at 20°C with shaking at 150 rpm. Next, the bacterial cells were harvested by centrifugation at 7,000 x g and 4°C for 30 min. The induced bacteria were resuspended in 5 mL of 10 mM sodium phosphate buffer, pH 7, containing 100 mM NaCl and 20 mM imidazole and then disrupted by sonication (4 ultrasound pulses of 15 s at output 3 in a Branson Sonifier 250 (Branson Instruments, Stanford, CT, US)). The resuspended cells were incubated on ice during the sonication procedure, and a 3 min cooling step was introduced between the ultrasound pulses. Then, the cell debris was removed by centrifugation (7,000 x g, 4°C and 30 min). For purification of the recombinant protein, aliquots of the lysate supernatant (1 mL) were mixed with nickel-nitrilotriacetic resin (Ni-NTA; Qiagen, Valencia, CA, US) for 1 h at 25°C with gentle end-to-end agitation. The unbound proteins were then removed via washes with 10 mM sodium phosphate buffer, pH 7, containing 100 mM NaCl and 20 mM imidazole (0.75 mL), and the solution was centrifuged (13,200 x g, 4°C, 1 min). Five wash steps were performed. Proteins bound to the resin were eluted by incubating them with 10 mM sodium phosphate buffer, pH 7, containing 100 mM NaCl and 500 mM imidazole (0.2 mL) for 30 min on ice. The mixture was then centrifuged (13,200 x g, 4°C, 1 min), and the supernatant was recovered. This protein sample was subjected to a buffer exchange step using a High Trap Desalting Column (GE HealthCare, Little Chalfont, UK). The protein samples were further purified using ion exchange chromatography with a MonoQ 5/50 column (GE HealthCare) and 20 mM HEPES, pH 7.0, and 20 mM HEPES, pH 7.0, buffer containing 1 M NaCl (flow rate: 1 mL/min). A salt gradient (0.2 to 0.8 M) was used to elute the recombinant proteins. Fractions (0.4 mL) collected in the elution step were used for bglTm detection based on its enzyme activity. SDS-PAGE [21] was used to verify the purification of the wild-type and mutant bglTm proteins. The protein concentration was determined by monitoring the absorption at 280 nm in the presence of 6 M guanidine hydrochloride prepared in 50 mM sodium phosphate buffer, pH 6.5. The extinction coefficients were calculated for the wild-type and mutant bglTm proteins [22, 23].

### Mutant design

**Fig 1:**
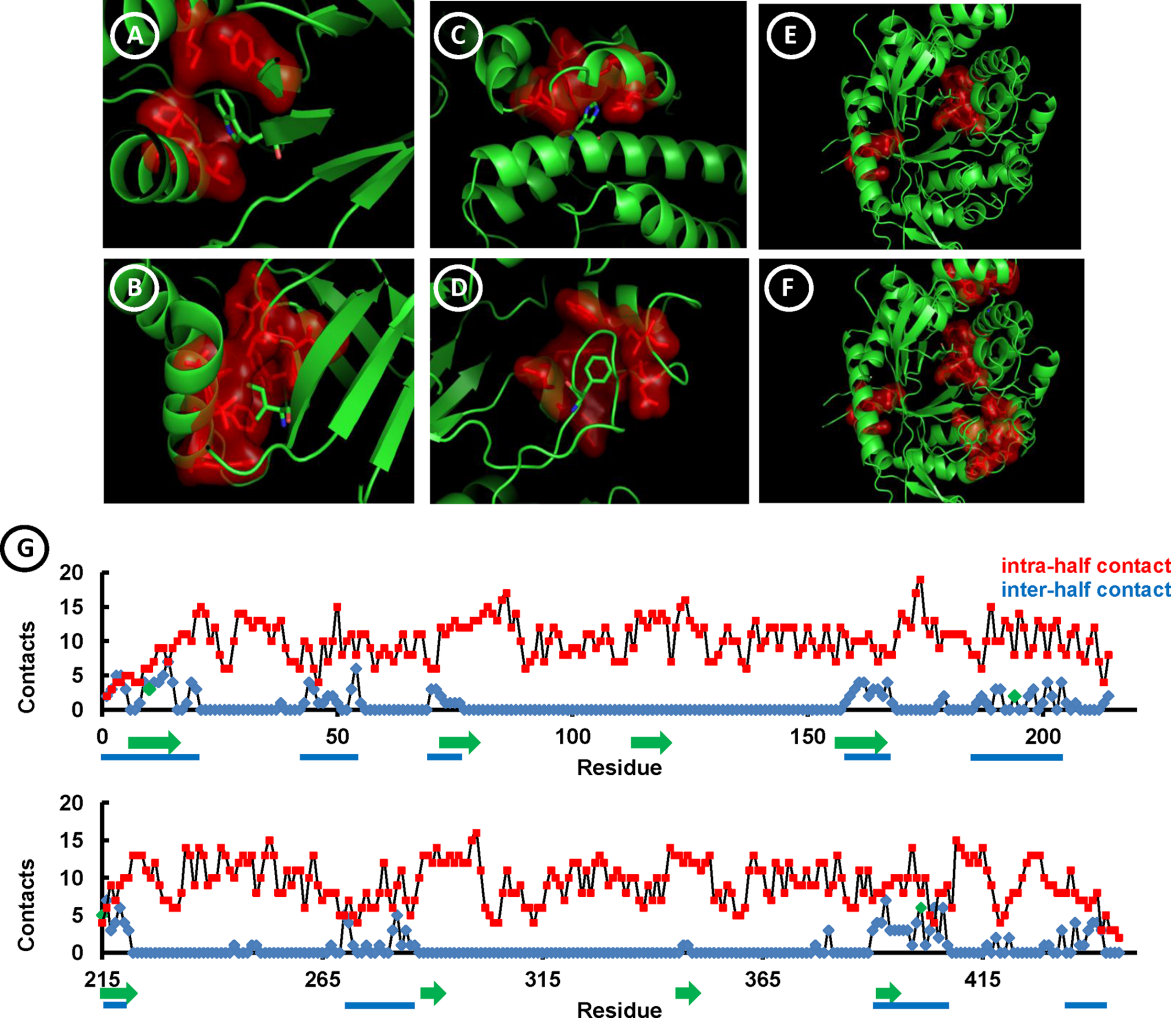
Distribution of mutations in the bglTm structure. Mutated residues (W12, H195, I217 and F404) are represented as sticks, whereas their contacting neighbors (distance cutoff: 5 Å) are shown as sticks covered by their molecular surface (in red). (A) Residue W12. (B) Residue I217. (C) Residue H195A. (D) Residue F404. (E) Mutant T1 contains two replacements, W12A and I217A. (F) Mutant T2 contains four replacements, W12A, H195A, I217A and F404A. (G) Noncovalent contacts formed by bglTm residues. Red coloring indicates intra-half contacts. Blue coloring indicates inter-half contacts. Green arrows represent the eight β-strands (in increasing order) that form the bglTm internal β-barrel. Blue bars indicate the protein segments that form the inter-half contacts. The number of contacts per residue was determined using the Protein Contact Map tool [24] available from the nanoHUB server. The cutoff distance for contacts is 8 Å between C_α_. The mutated residues (W12, H195, I217 and F404) are indicated by green dots.

The bglTm crystallographic structure (1OIN) was uploaded into the Contact Map Tool available at the nanoHUB server [24]. The contact cutoff was set to 8 Å between C_α_. The number of contacts per residue was calculated and sorted into intra-half and inter-half contacts. The bglTm halves (residues 1 to 215 and 216 to 446, respectively) were defined according to the number of residues and content of the secondary structure elements (4 contiguous β strands; Fig 1). Regions containing a higher number of inter-half contacts were initially identified. The structural details of these regions were visualized using PyMOL software (The PyMOL Molecular Graphics System, version 1.8 Schrödinger, LLC). Thus, within the inter-half contact regions, residues from one half containing side chains that inserted into a group of neighbors in the opposite half were selected for replacement with alanine. Neighboring residues were defined as residues that present any atom within 5 Å from another residue. Mutations were designed after considering that alanine substitutions might disrupt the contacts formed by residue side chains. In addition to these criteria, mutation sites were designed to be uniformly distributed in the bglTm structure. Finally, the same number of replacements was introduced in each bglTm half. Thus, the T1 mutant contained one replacement per half (W12A and I217A), whereas the T2 mutant contained two substitutions per half (W12A –H195A and I217A –F404A).

### Determination of the tryptophan fluorescence spectra and quenching by acrylamide

The fluorescence spectra of the purified protein samples were collected at 30°C using an F-4500 Hitachi spectrofluorimeter (Hitachi, Tokyo, Japan). Samples were excited at 295 nm, and the emission spectra were recorded in the range of 305 to 400 nm. The scanning speed was 12 nm/min, the slit widths were set to 5 nm (emission and excitation), and the integration time was 1 s. Fluorescence quenching was performed using different concentrations of acrylamide prepared in 10 mM sodium phosphate buffer, pH 6.0. The quenching effect was evaluated by plotting the ratio of the maximum fluorescence (F) in the presence of acrylamide to the maximum fluorescence in the absence (F_0_) of acrylamide *versus* the acrylamide concentration. The Stern-Volmer constant (K_sv_), which indicates the extent of tryptophan exposure to the solvent, was calculated from the slopes of the F/F_0_ *versus* [acrylamide] graphs [25, 26].

### Determination of the enzymatic activity and kinetic parameters

β-Glucosidase activity was detected using *p*-nitrophenyl β-D-glucopyranoside (NPβGlc) and *p*-nitrophenyl β-D-fucopyranoside (NPβFuc) as substrates. Both substrates were prepared in 50 mM sodium citrate–sodium phosphate buffer, pH 6.0. Assays were conducted at 30°C. Reactions were terminated by the addition of 250 mM sodium carbonate, pH 11, and the product (*p*-nitrophenolate) was detected by monitoring the absorbance at 415 nm [27]. Substrate and enzyme samples were incubated for different times. The initial rate of product formation was calculated from the linear [P] *versus* time plots. For the determination of the kinetic parameters, at least 10 different substrate concentrations were used in the enzyme assays. The initial rates and substrate concentrations were fitted using the Michaelis-Menten equation in Enzfitter software [28]. Alternatively, when decreasing rates were observed at high substrate concentrations, suggesting the occurrence of transglycosylation reactions, the equation *v*_0_ = *V*_max_*[S] / {*K*_m_ + ([S] * (1 + (*K*_m_ / *K*_mB_) + ([S] / *K*_mB_))} was applied to the fitting process. In the equation, *K*_mB_ represents the binding of a second substrate to form a complex that proceeds through the transglycosylation reaction pathway [29].

### Thermal denaturation assessed by monitoring enzyme activity

Samples of the wild-type and mutant bglTm proteins were separately incubated with 50 mM sodium citrate–sodium phosphate buffer, pH 6.0, at 47, 70, 75 and 80°C. Aliquots were removed after different periods of time (0 to 40 min) and stored on ice. The remaining enzyme activity of those samples was then determined at 30°C using 8 mM NPβGlc (prepared in 50 mM sodium citrate–sodium phosphate buffer, pH 6.0). These experiments, which depend on catalytic activity measurements, were performed at pH 6 because this condition favors the activity detection. The kinetic profile of thermal inactivation was analyzed by plotting the logarithm of the relative remaining activity *versus* the incubation time at the higher temperature (47, 70, 75 and 80°C) [30].

### Thermal denaturation assessed using circular dichroism (CD) spectroscopy

The bglTm samples (10 μM) were prepared in 10 mM potassium phosphate buffer, pH 7. Measurement noise was reduced in this buffer. Samples were conditioned in rectangular quartz cuvettes with path length of 0.1 cm (NGS Precision Cells Inc., Farmingdale, NY, US) to collect the CD data. The CD spectra were collected at two different wavelengths, 222 and 208 nm, using a Jasco J-815 spectropolarimeter. The temperature of the sample was progressively increased from 20 to 90°C at a rate of 0.5°C per minute using a Peltier system. As the sample reached each target temperature, it was allowed to equilibrate for 5 s [31].

### Thermal denaturation assessed using differential scanning fluorimetry (DSF)

Samples of bglTm (1.6 μg) prepared in 10 mM potassium phosphate buffer, pH 7 (22.5 μL) were mixed with Sypro Orange dye (2.5 μL; Sigma-Aldrich, St. Louis, MO, US). This mixture was subjected to an incremental temperature gradient (25 to 95°C at a rate of 0.1%) in a Real Time PCR 7500 system (Life Technologies, Carlsbad, CA, USA) set in the melting temperature mode. The fluorescence data were detected using filter 2 (approximately 550 nm). The melting temperature (*T*_m_) was determined by plotting the maximum value of the first derivative of the fluorescence *versus* temperature curve [31, 32].

### Denaturation with guanidine hydrochloride

The fluorescence spectra of the wild-type and mutant bglTm proteins were recorded (as described above) in the presence of different concentrations of guanidine hydrochloride (0 to 7 M) prepared in 50 mM sodium citrate–sodium phosphate buffer, pH 6.0. Relative fluorescence was determined using the intensity at the maximum emission peak (λ_max_) of the spectrum in the presence of 7 M guanidine hydrochloride as a reference. Subsequently, the relative fluorescence at the λ_max_ for each spectrum was plotted as a function of the guanidine hydrochloride concentration. The nonlinear least squares method [33] was used to directly fit the relative fluorescence data (RF) using the equation RF = {(b_1_ + b_2_[G]) + (a—(b_1_ + b_2_[G]))} / {1 + exp^(([G]—c50) / d)^}, where [G] is the guanidine hydrochloride concentration, *c*_50_ is the transition concentration at which the native and unfolded protein concentrations are the same, b_1_ + b_2_[G] describes the relative baseline fluorescence in the post-transition region and “a” is the relative baseline fluorescence in the pre-transition region [34–36]. The *m*-values were directly derived from the fitting process, as the equation constant *d* corresponds to *RT*/m [34]. The c_50_ parameter was also directly derived from the fitting process. Finally, the protein stability in the absence of denaturant, ΔG_H20_, was calculated using the equation ΔG_unfold_ = ΔG_H20_ –m[G]. Thus, if [G] = c_50_, then ΔG_unfold_ = 0 and the previous equation is rearranged to ΔG_H20_ = m.c_50_, as described previously [34–36]. Origin 2017 software (Origin Lab, Northampton, MA, US) was used for the nonlinear fitting process.

## Results and discussion

According to previous experimental evidence [4–8], half-barrels exist as self-contained units within the (β/α)_8_ barrels. Theoretically, this hypothesis should be valid for any type of (β/α)_8_ barrel, independent of its size and internal symmetry. We studied a large (β/α)_8_ barrel protein, the β-glucosidase bglTm, to test this hypothesis. We aimed to determine the individual properties of the half-barrels and obtain evidence of their theoretical coexistence within the (β/α)_8_ barrel; thus, we produced mutants containing disruptions of noncovalent interactions between the bglTm halves.

Mutant T1 contained the W12A and I217A mutations, whereas mutant T2 contained two additional mutations, H195A and F404A (Fig 1). Based on the contact map of bglTm, these residues are located in segments that formed a relatively higher number of inter-half contacts (Fig 1). Interestingly, the inter-half contacts were the minor contacts and were concentrated in 4 and 5 segments on each half, respectively. Indeed, residues generally formed contacts within the halves (Fig 1). Additionally, the inter-half contacting segments 1, 4, 6 and 8 coincided with β-strands 1, 4, 5 and 8 (Fig 1), as expected based on the hydrogen-bonding network predicted for β-barrels, whose halves are joined between β-strand pairs 1–8 and 4–5 [37, 38]. Mutant T1 contained mutations that were symmetrically distributed in the β-barrel joints within β-strands 1 and 5. Mutant T2 contained two additional mutations in the α-helices located outside the barrel (Fig 1). Finally, the side chains of W12, H195, I217 and F404 were enveloped by a group of residues from the opposite half (marked in red), with which they formed noncovalent contacts (Fig 1). Briefly, the T1 and T2 mutations were intended to disrupt contacts in the half joints. Based on the bglTm structure, inter-half contacts with 15 residues would be theoretically perturbed by the two mutations introduced in T1, whereas the inter-half contacts with 30 residues would be disturbed by the four replacements in T2.

**Fig 2:**
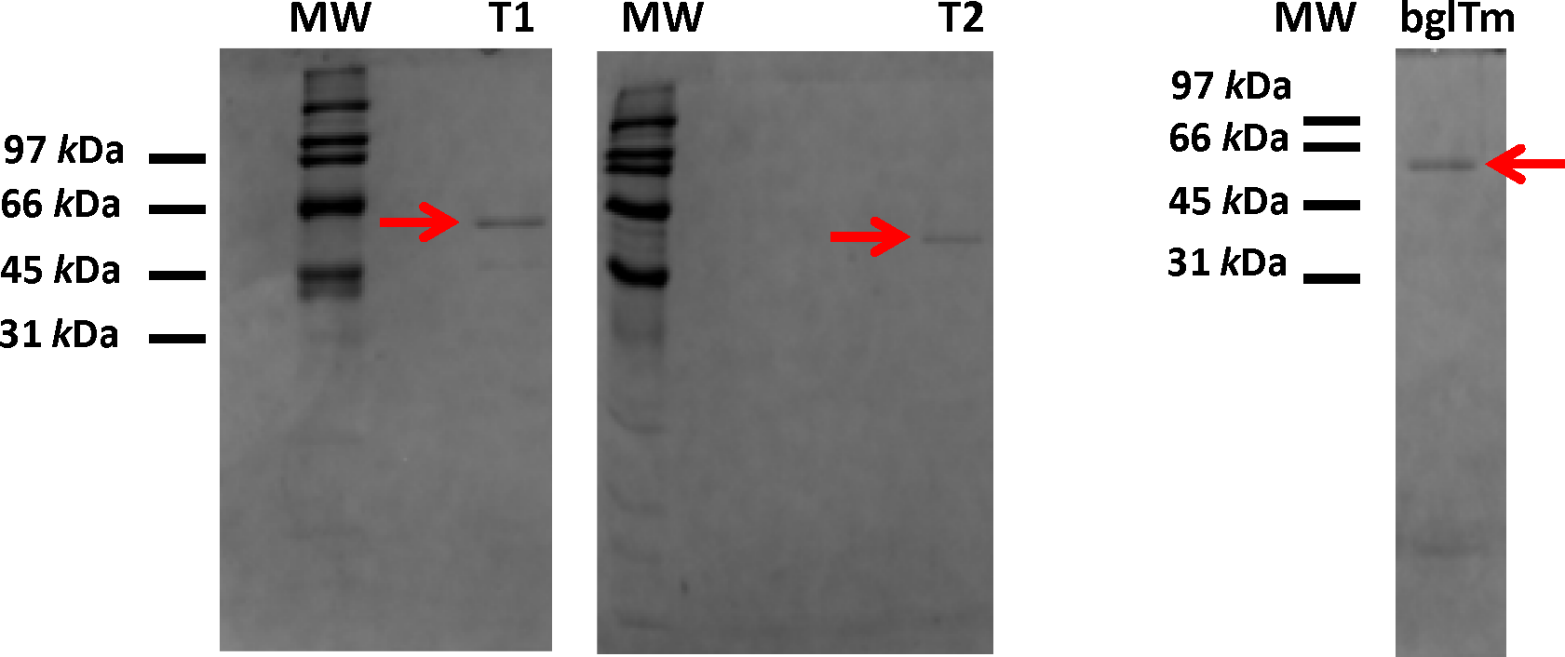
SDS-PAGE analysis of the purified wild-type and mutant bglTm (T1 and T2) proteins. Gels were stained with Coomassie Blue R. Arrows indicate the purified proteins. MW, molecular weight marker.

The wild-type bglTm protein and mutants T1 and T2 were produced as recombinant proteins in *E*. *coli* BL21(DE3) and purified with Ni-NTA resin and ion exchange chromatography (Fig 2). The estimated relative molecular weights (61 kD for wild-type bglTm, T1 and T2 after subtracting the fusion peptide weight) were compatible with the expected molecular weights based on the protein sequence [18].

**Fig 3:**
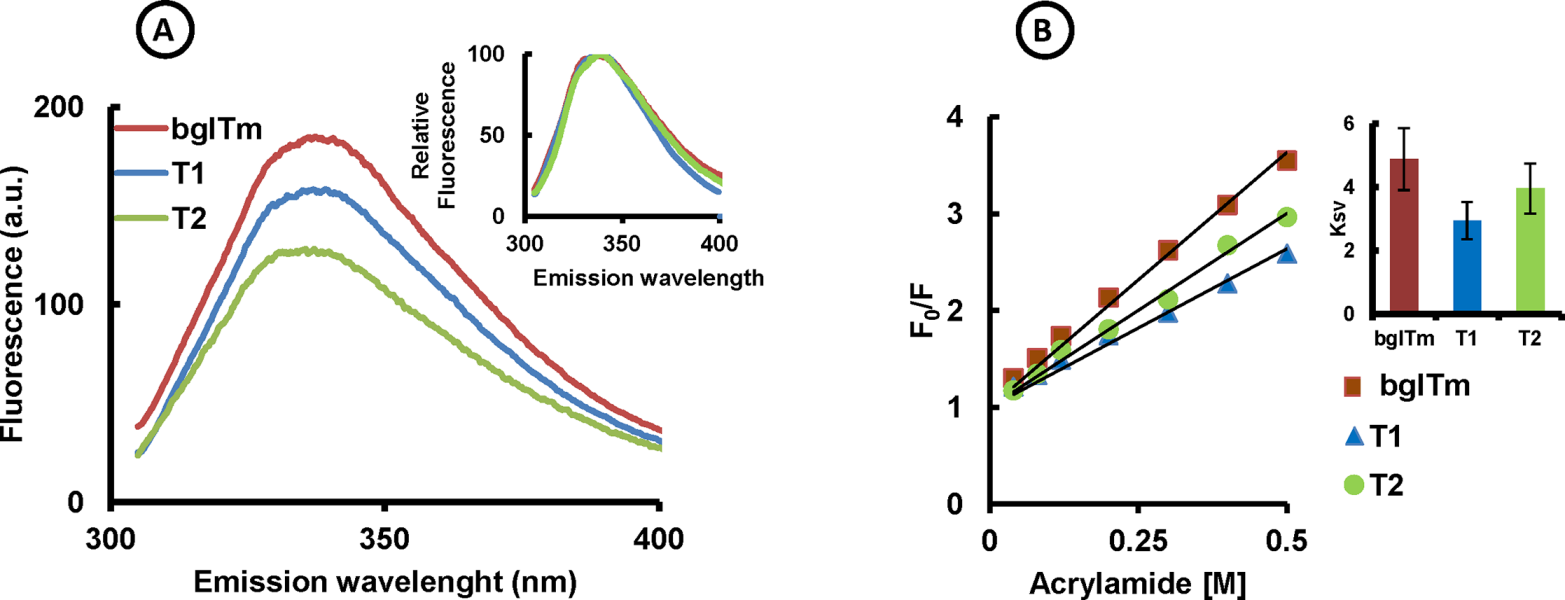
Fluorescence spectra and acrylamide quenching of the wild-type and mutant bglTm proteins. (A) Intrinsic fluorescence spectra. Red, wild-type bglTm; blue, T1 mutant; green, T2 mutant. The samples were excited at 295 nm. Insert: relative fluorescence spectra. (B) Effect of acrylamide on bglTm fluorescence. Red, wild-type bglTm; blue, T1 mutant; green, T2 mutant. F, fluorescence in the presence of acrylamide; F_0_, fluorescence in the absence of acrylamide. Fluorescence readings were measured at the wavelength (λ_max_) with the highest emission in the absence of acrylamide. Insert: K_sv_ parameters with respective deviations for wild-type and mutant bglTm proteins.

**Fig 4:**
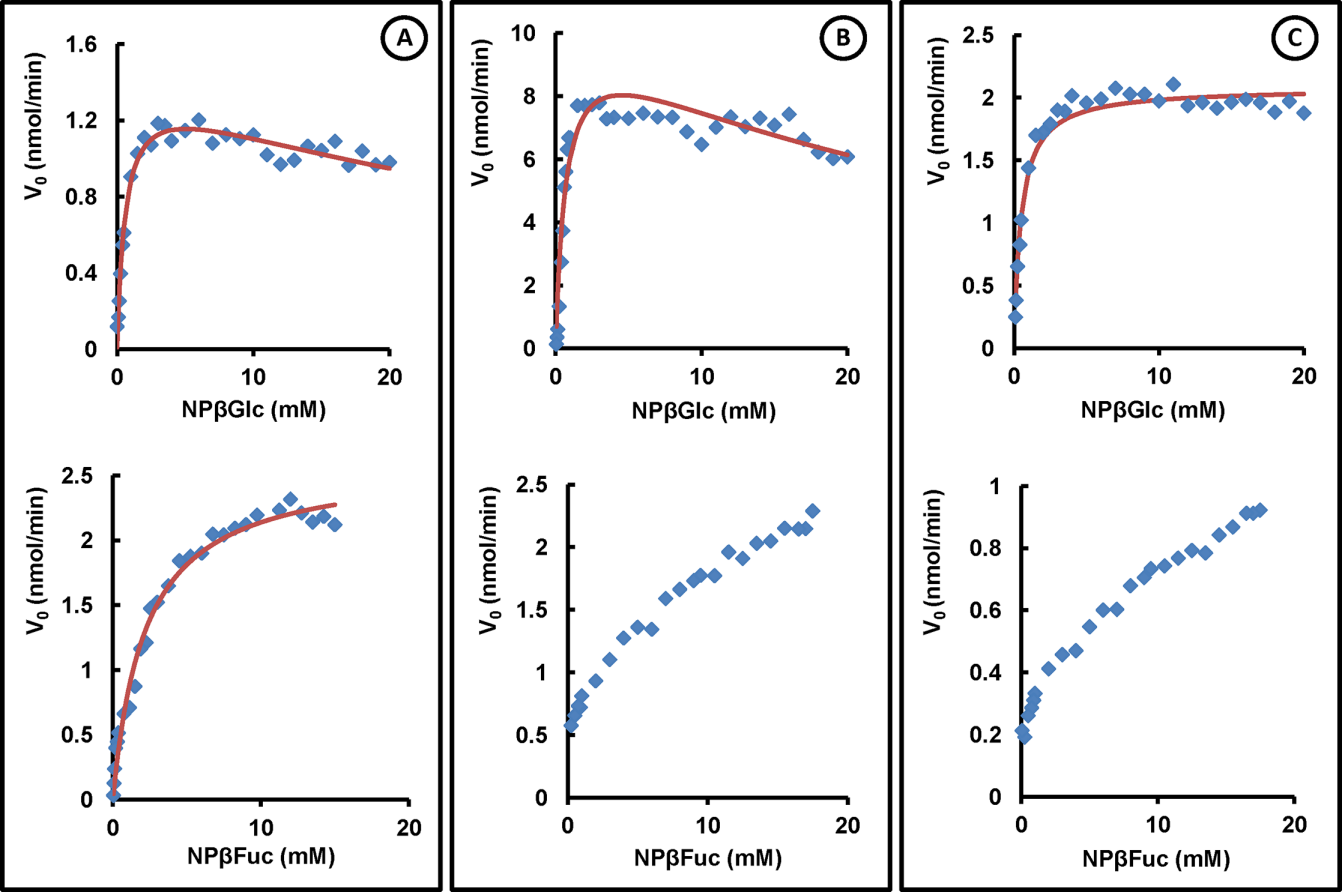
Effect of the substrate concentration on the initial rate (nmol/min) of the hydrolysis reaction catalyzed by the wild-type and mutant bglTm proteins. (A) Wild-type bglTm. (B) Mutant T1. (C) Mutant T2. The tested substrates were *p*-nitrophenyl β-glucopyranoside (NPβGlc) and *p*-nitrophenyl β-fucopyranoside (NPβFuc). Lines indicate the best fits of the experimental data (dots) using the appropriate kinetic equation (see the Materials and Methods for details).

The tryptophan fluorescence spectra suggested that these proteins were folded. In fact, according to the results of our acrylamide quenching experiments, the solvent exposure of the tryptophan residues in T1 and T2 (K_sv_ = 2.9 ± 0.6 and 3.9 ± 0.9, respectively) was similar to the wild-type bglTm protein (K_sv_ = 4.9 ± 0.9) if we considered their respective deviations (Fig 3). Finally, mutants T1 and T2 were active toward the same substrates (NPβGlc and NPβFuc) that were hydrolyzed by the wild-type bglTm protein (Fig 4). Because the active site of bglTm is formed by residues Q20, H121, N160, E161, Y295, E351, W398, E405 and W406 [18] from both halves of the barrel, the observed catalytic activity also provides evidence that T1 and T2 were native proteins, despite the disruption of contacts between their halves. Notably, the wild-type bglTm and T1 proteins did not follow classical Michaelis-Menten kinetics for the hydrolysis of NPβGlc, suggesting the probable occurrence of transglycosylation reactions catalyzed by these enzymes [29, 39]. However, these reactions were not observed for the mutant T2 protein. After considering the appropriate kinetic model, T1 and T2 presented the same *K*_m_ toward NPβGlc (*K*_m_ = 0.7 ± 0.1 mM and 0.48 ± 0.04 mM, respectively) as the wild-type bglTm protein (*K*_m_ = 0.63 ± 0.07 mM). Moreover, mutants T1 and T2 had an increased *K*_m_ toward NPβFuc, because a rate plateau at high substrate concentration was not observed for these enzymes as was observed for the wild-type bglTm protein (Fig 4). Thus, T1 and T2 were native proteins with structured active sites, which otherwise presented architectural differences that altered the substrate binding and catalysis.

**Fig 5:**
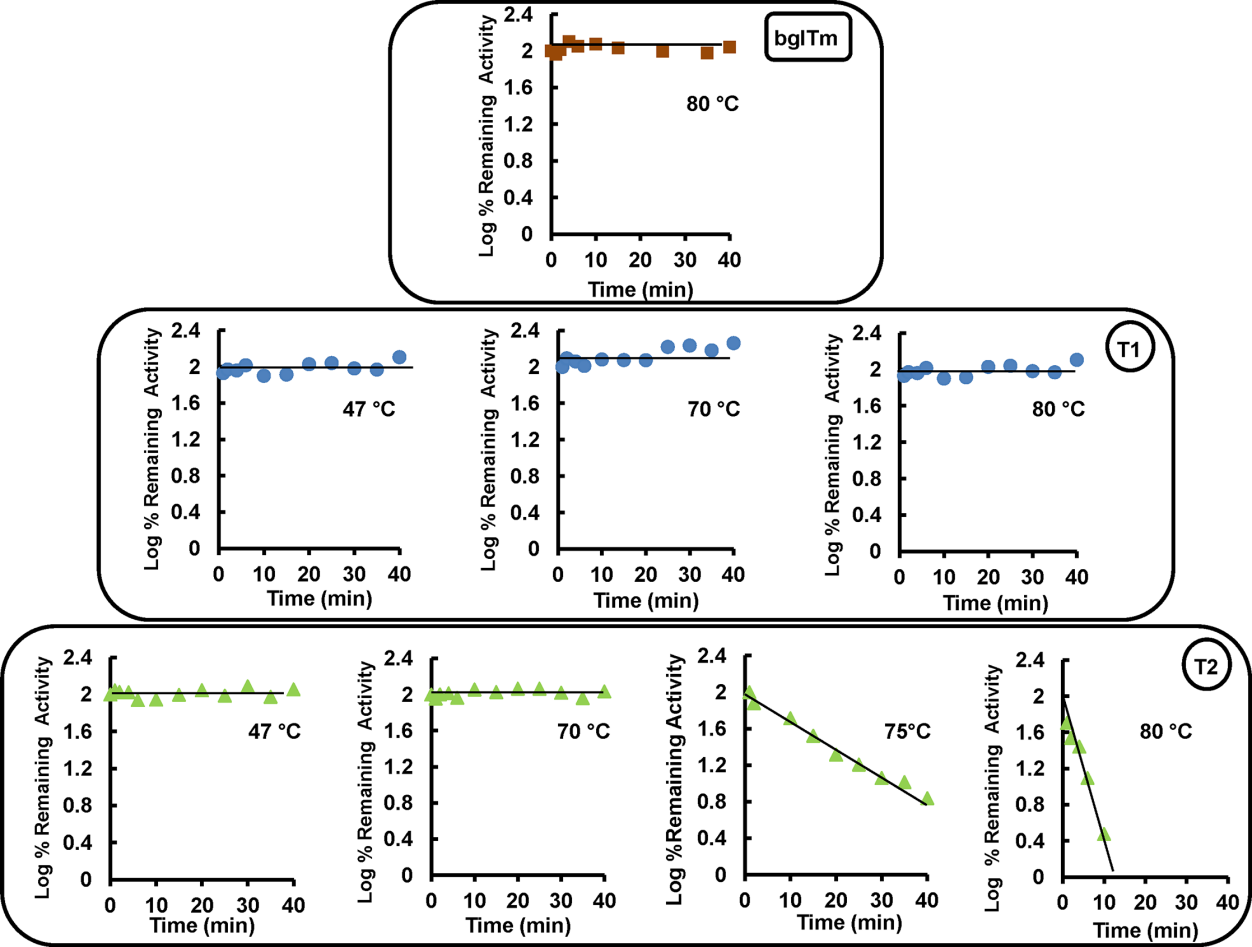
Thermal inactivation of the wild-type and mutant bglTm proteins. Red, wild-type bglTm; blue, T1 mutant; green, T2 mutant. The stability of the wild-type bglTm protein was probed at 80°C. The stability of the mutant T1 protein was probed at 47, 70 and 80°C, and the stability of the mutant T2 protein was probed at 47, 70, 75 and 80°C. Enzyme samples were incubated at the indicated temperatures for different times (indicated in the plots), and the remaining enzyme activity in each sample was determined at 30°C using 8 mM NPβGlc prepared in 50 mM sodium citrate–sodium phosphate buffer, pH 6.0. Linear inactivation profiles, i.e., first-order kinetics, indicate that the protein inactivates as a single element.

Because the mutant proteins were properly folded, we probed their thermal stability. The wild-type bglTm protein was stable at temperatures up to 80°C, showing no decrease in the remaining relative activity (Fig 5). The same result was observed for the T1 mutant. However, the T2 mutant was inactivated at 75 and 80°C (Fig 5). The inactivation of T2 is a clear indication that the disruption of the contacts between the barrel halves destabilized this mutant. Importantly, based on the observed linear inactivation pattern, i.e., first-order kinetics, T2 was clearly inactivated as a single unit. Hence, we did not obtain evidence of independent inactivation of each barrel half in T2, which would produce a curvilinear inactivation pattern. Thus, T2 behaved as a single domain, despite the disruption of its half-barrel contacts.

**Fig 6:**
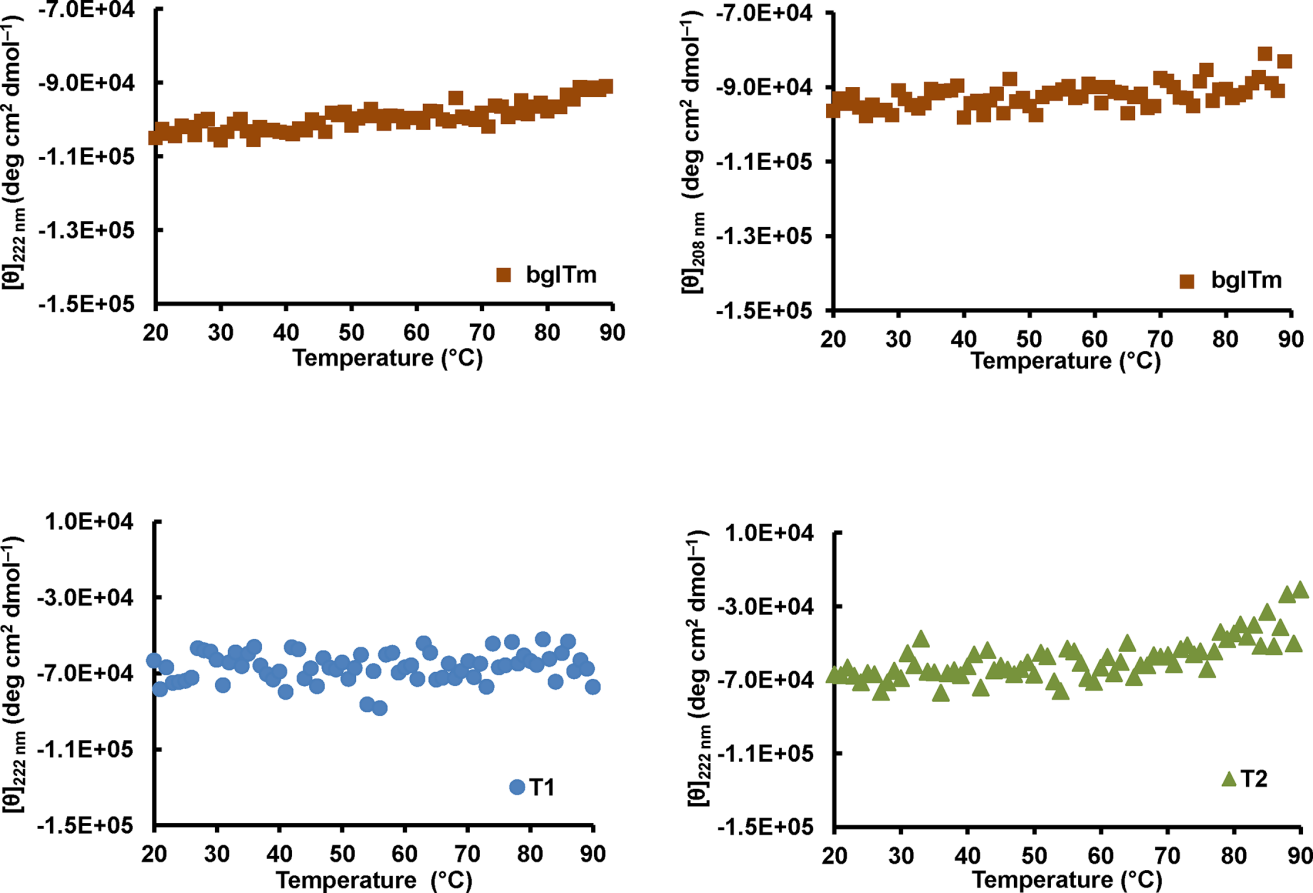
Thermal unfolding of the wild-type and mutant bglTm proteins, as monitored using CD spectroscopy. Red, wild-type bglTm; blue, T1 mutant; green, T2 mutant. The CD spectra were recorded at 222 nm and 208 nm for wild-type bglTm and only at 222 nm for T1 and T2. The samples were prepared in 10 mM potassium phosphate buffer, pH 7.

**Fig 7:**
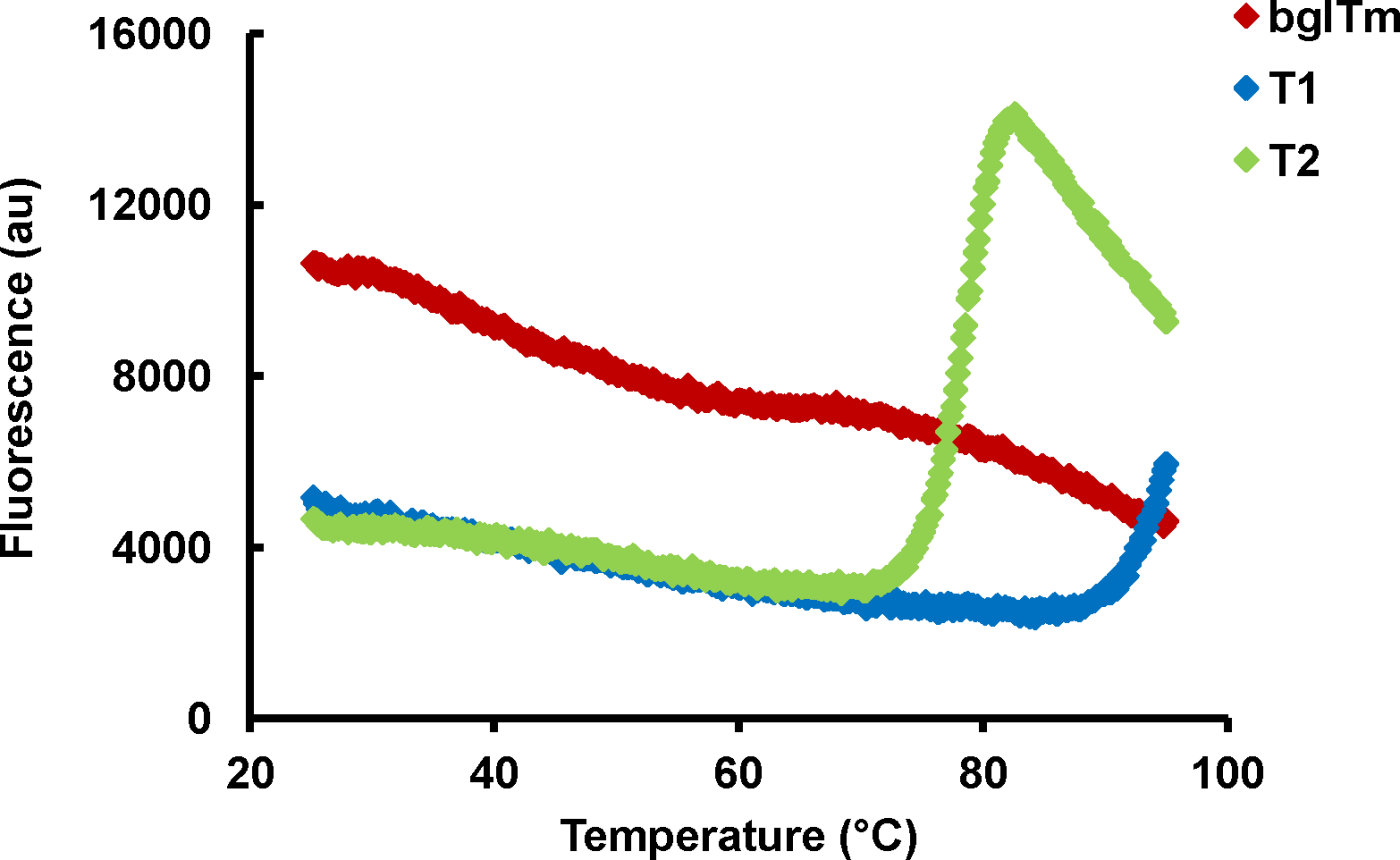
Thermal unfolding of the wild-type and mutant bglTm proteins, as monitored by DSF. Red, wild-type bglTm; blue, T1 mutant; green, T2 mutant. Samples were prepared in 10 mM potassium phosphate buffer, pH 7.

The analysis of the structural thermal stability of the proteins using CD spectroscopy and DSF experiments showed that wild-type bglTm was stable at temperatures up to 90°C and showed no thermal transition to a non-structured state. The same result was also observed for T1 (Figs 6 and 7), whereas a clear thermal transition (with a single *T*_m_ = 82°C) was present in the data for T2 from the DSF experiments (Fig 7). This result was consistent with the thermal inactivation at 80°C observed for T2 (Fig 5), thus confirming that this mutant was less stable. Again, we did not observe independent denaturation of each T2 half-barrel, which should produce a double transition (Fig 7).

**Fig 8:**
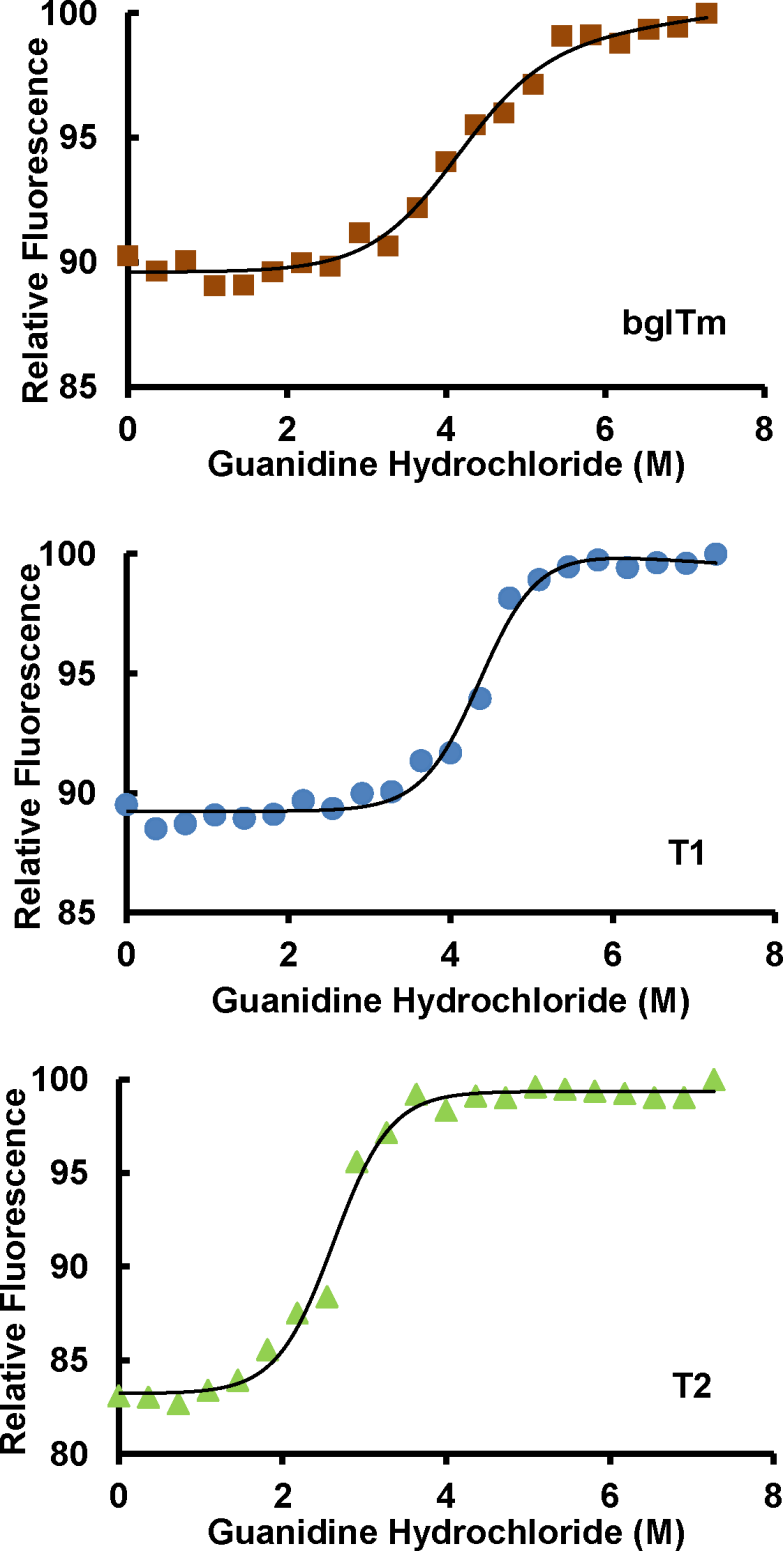
Denaturation of the wild-type and mutant bglTm proteins with guanidine hydrochloride. Red, wild-type bglTm; blue, T1 mutant; green, T2 mutant. The fluorescence spectra were recorded in the presence of different concentrations of guanidine hydrochloride prepared in 50 mM sodium citrate–sodium phosphate buffer, pH 6.0.

The structural stability of the wild-type and mutant bglTm proteins was also probed using chemical denaturation with guanidine hydrochloride (Fig 8). Based on the c_50_ and Δ*G*_H20_ values, T2 was less stable than T1 and wild-type bglTm (Table 1). Thus, the T2 mutant had low thermal and chemical stabilities. These three enzymes exhibited a simple two-state denaturation process with a single transition concentration (*c*_50_). Indeed, we did not observe a combination of two independent denaturation curves that would putatively correspond to individual halves. Importantly, based on the *m*-value (Table 1), the denaturation of bglTm, T1 and T2 notably exhibited the same cooperativity. The decrease in the halves’ mutual influences should have changed the denaturation cooperativity in the mutants. Nevertheless, despite the disruption of the contacts between their halves, T1 and T2 denatured as single units, similar to the wild-type bglTm protein. Moreover, the equilibrium and kinetic analysis of the TIM unfolding induced by guanidine hydrochloride have also revealed that it denatures as a single unit [7].

**Table 1:** Parameters for the denaturation of the wild-type and mutant bglTm proteins with guanidine hydrochloride.

Protein	c_50_ (M)	m (kcal/mol/M)	Δ*G*_H20_ (kcal/mol)
bglTm	4.3 ± 0.2	1.2 ± 0.2	5.1 ± 0.5
T1	4.5 ± 0.2	1.4 ± 0.2	6.3 ± 0.5
T2	2.4 ± 0.2	1.6 ± 0.2	3.8 ± 0.4

Protein denaturation was measured by monitoring the tryptophan fluorescence. N = 2. See Materials and Methods for details.

Briefly, based on the data presented here, the mutations indeed disrupted the contacts between bglTm halves, particularly in T2, which exhibited low thermal (Figs 5–7) and chemical stability (Fig 8). Nonetheless, we did not obtain evidence of the presence of individual half-barrel domains in the denaturation and inactivation of T2 and T1. Hence, the (β/α)_8_ barrels are likely each a single-domain protein. However, this conclusion conflicts with previously published evolutionary and thermodynamic data [5–14; 16]. Thus, a consensus interpretation is that the half-barrel domains of bglTm are actually alike, although they do not show any remarkable sequence similarity. Hence, even if the domains are partially isolated, their unfolding behavior overlaps.

In conclusion, based on our data, the halves that form bglTm are thermodynamically equivalent in terms of their thermal and chemical stability. Hence, their individual contributions to (β/α)_8_ barrel unfolding cannot be disentangled.

## Abbreviations

bglTm: β-glucosidase from *Thermotoga maritima*
NPβGlc: *p*-nitrophenyl β-D-glucopyranoside
NPβFuc: *p*-nitrophenyl β-D-fucopyranoside
